# Motives matter: The psychological experience of ostracizing among sources

**DOI:** 10.1371/journal.pone.0303510

**Published:** 2024-05-31

**Authors:** Rose Iannuzzelli, Karen Gonsalkorale, Lisa A. Williams

**Affiliations:** 1 School of Psychology, The University of Sydney, Sydney, Australia; 2 School of Psychology, The University of New South Wales, Sydney, Australia; St John’s University, UNITED STATES

## Abstract

Individuals ostracize others for myriad reasons, yet the influence of those reasons on the psychological experience of ostracizing is yet unknown. Two studies aimed to determine the emotional and behavioral sequelae of ostracizing for different motives, directly comparing punitive to defensive motives. We focused our examination on a suite of emotions expected to arise as a function of (1) the situations that give rise to ostracizing for punitive and defensive reasons (anger, fear, anxiety, and sadness) and (2) the act of ostracizing itself (i.e., pride and guilt). The research employed a novel paradigm to induce the experience of ostracizing for defensive or punitive motives. Study 1 (*N* = 372) investigated sources’ experienced emotion as a function of motive. Study 2 (*N* = 743) expanded consideration to behavioral intentions, including intentions to continue ostracizing and to recruit others to join in ostracizing the target. Across both studies and supported by an internal meta-analysis, ostracizing for defensive reasons was associated with higher levels of guilt, fear, and anxiety, and lower levels of anger, compared to ostracizing for punitive reasons. Neither sadness nor positive emotion (pride or happiness) differed significantly according to motive in either study. Moreover, guilt and anger mediated the impact of motive on intentions to continue ostracizing and recruit others to join them in ostracizing. To the extent that punitive sources experienced anger relative to defensive sources, they expressed greater intentions to continue ostracizing the target and to recruit others to join in ostracizing the target. To the extent that defensive sources experienced guilt relative to punitive sources, they reported reduced intentions to continue ostracizing the target. Findings add to a growing literature on ostracism sources, and highlight the mediating role of sources’ emotion in guiding future actions.

## Introduction

Ostracism, the act of being excluded and ignored, is ubiquitous across time, culture, and social context [1]. Most individuals will both be ostracized (i.e., be the ‘target’ of ostracism) and will ostracize another (i.e., be the ‘source’ of ostracism) at some point in their lives [2]. A rich history of social psychological research has examined consequences of being the target of ostracism (see review [3]), but only recently has research turned focus to the experiences of sources (e.g. [4–9]). In this expanding field, it is yet unclear how sources’ motives for ostracizing might determine the psychological experience of engaging in this social act. The current studies aimed to determine the emotional and behavioral sequelae of ostracizing for different motives.

Theoretical models of ostracism emphasize the specific motive driving ostracism as key for understanding both target and source experience [10–13]. Motives include ostracizing to punish the target for a perceived wrongdoing (i.e., punitive motives), ostracizing to protect oneself from the target or to preclude escalation (i.e., defensive motives), ostracizing when dictated by situational social norms (i.e., role-prescribed motives), and ostracizing because the target’s presence was not noticed in the first place (i.e., oblivious motives; [11, 12, 14, 15]). The current research focuses on the first two of these.

Punitive motives dominate real life instances of ostracism [2, 16, 17]. Mirroring this pattern, laboratory-based experiments have focused predominantly on inculcating punitive motives (see [4] for a review). Namely, in group-based game play paradigms, potential targets of ostracism are programmed to be burdensome to participants’ patience and/or performance outcomes [18–23]. In these paradigms, participants exclude slow or poor-performing players and desire less future interaction with them compared to non-burdensome players. Parallel findings have been documented in the context of social media [24].

Defensive motives stem from a source’s desire to protect themselves and can aim to pre-empt ostracism or further escalation in a conflict situation. This definition aligns with that provided by Zadro and colleagues [25] and highlights that defensive motives can be both pre-emptive and preventative in their basis, including motives related to ‘conflict avoidance’ and ‘timeout’ [17]. This inclusive definition spans the many forms that self-protection motives might take.

Punitive and defensive motives share some characteristics, but are also putatively distinct. Both punitive and defensive motives are autonomous [6] and are commonly reported reasons for engaging in ostracism in everyday life [2, 16, 17]. Yet, they differ in key characteristics. Henle and colleagues’ [15] systematic review of the literature on motives for ostracism highlighted key differences between these two core motives. In their view, punitive motives are driven by the desire to bring the behavior of substandard group members in alignment with group norms, with the ultimate aim of benefitting group cohesion and performance. The core driver of defensive motives, on the other hand, is self-oriented rather than group-oriented. Defensive sources aim to shield themselves from actual or potential harm and/or to distance themselves from others who might cause them to be ostracized. The key psychological processes underpinning defensive motives are ego protection and self-enhancement. Given the fundamental differences between these two motives, it is perhaps surprising that the experience and outcomes for defensive and punitive sources have not to date been compared empirically. Guided by the premise that focusing on motives provides a useful framework for organizing and understanding the experience of those involved in ostracism episodes [26], we compared the psychological experience of sources motivated by either punitive or defensive reasons.

In this research, we focused primarily on sources’ emotional experience. Sources’ emotions not only may guide future action in relation to the ostracism episode (as tested in Study 2), but also represent psychological states that might be intervened upon in order to shorten or end ostracism. A focus on emotions also aligns with Henle and colleagues’ [15] call for more research on the impact of source motives on negative emotions. As such, it is of theoretical as well as practical import to understand how sources’ motives may give rise to the emotions they experience while ostracizing.

We focused our examination on a suite of emotions expected to arise as a function of (1) the situations that give rise to ostracizing for punitive and defensive reasons (anger, fear, anxiety, and sadness) and (2) the act of ostracizing itself (i.e., pride and guilt). Our selection of emotions to focus upon was guided by appraisal theories of emotion [27–30], in light of the situations held to give rise to punitive and defensive motives for ostracism.

Punitive motives stem from feeling wronged by the target–either personally or on behalf of one’s group–where ostracizing is a form of retaliation against perceived injustice [11, 12, 14, 15]). In a similar vein, anger is driven by appraisals that someone else is to blame for an offensive injustice to the self [31–33]. As such, the sense of having been wronged that is characteristic of situations that give rise to punitive motives should elicit relatively high levels of anger. Indeed, previous research has found that individuals report increased anger when ostracizing for punitive reasons relative to ostracizing for other reasons [2, 16, 34]. In line with these findings, we predicted that sources motivated by punitive reasons would report higher levels of anger than sources motivated by defensive reasons.

Defensive motives, on the other hand, stem primarily from a pair of concerns: those regarding a sense of personal threat and those in relation to future negative events, such as fears about the situation getting out of hand. In these cases, ostracizing is a means of self-protection [12, 14]. Often, defensive motives stem from a lack of personal control in the situation [12, 14], which can produce a sense of helplessness [35]. Fear and anxiety are elicited by appraisals of threat that are uncertain in nature and over which the self has little control [29, 30]. While sadness shares the theme of low personal control with fear and anxiety, sadness stems from negative outcomes that are relatively certain rather than uncertain in nature [29, 30]. This gives rise to a key characteristic of sadness: helplessness [33]. Past work has not, to our knowledge, specified the certainty characteristics associated with different source motives. As such, it may be the case that fear, anxiety, and sadness are elicited to varying degrees across situations that give rise to defensive motives according to associated appraisals regarding the uncertainty of future events. In any case, we predicted that sources motivated by defensive reasons would report higher levels of all three of these emotions than sources motivated by punitive reasons due to the core themes of threat and low personal control.

Emotional experiences of sources may also arise from the act of ostracizing itself. Here, the self-conscious emotions of guilt and pride are relevant. Both guilt and pride stem from appraisals that one’s past or ongoing actions are controllable (i.e., can be wilfully chosen) and variable across time (i.e., temporally unstable rather than something innate about the self; [36]). Guilt and pride are differentiated in terms of the degree to which the outcomes or behavior in question are congruent with current identity goals. For example, pride arises from identity goal congruent acts, and guilt from identity goal incongruent acts [36].

Broadly, engaging in ostracism is likely incongruent with identity goals, given that most individuals hold themselves to be relatively communal (i.e., kind, friendly, sociable; [37]; see also [6]). However, defensive and punitive motives may give rise to differential levels of pride and guilt. Punitive ostracism, with its overarching goal of retaliation, is likely to activate agentic identity goals (i.e., to be dominant, vindictive, aggressive) for sources. The congruence between punitively ostracizing and agentic goals should thus increase pride and decrease guilt. This is in line with previous research finding that individuals ostracizing for punitive reasons report less guilt compared to ostracizing for other reasons [38]. Thus, we predicted that sources motivated by punitive reasons would feel more pride and less guilt about ostracizing than sources motivated by defensive reasons.

Given that pride was the only positive emotion in the suite of theoretically-relevant emotions we identified, it is possible that any observed differences could be driven by broad positive affect rather than differences in pride specifically (for similar logic, see [39]). As such, we additionally assessed the impact of ostracizing for different motives on experienced happiness to determine the degree to which observed differences in pride were unique to pride or may be attributable to general positive valence. As there was no theoretical reason to expect differences in happiness according to source motive, we did not expect levels of happiness to differ according to condition.

A pilot study, reported in S1 File, established the efficacy of a paradigm in which participants recall or imagine themselves ostracizing for punitive or defensive motives. Study 1 deployed this paradigm to investigate sources’ emotional experiences while ostracizing. Study 2 further examined behavioral intentions to continue ostracizing and to recruit others to also ostracize, and whether the impact of motive on such intentions is mediated by experienced emotion. All study materials and data, as well as preregistrations of Studies 1 and 2, can be found on the project Open Science Framework (OSF) site: https://osf.io/632ye/. We report how we determined our sample sizes, all data exclusions (if any), all manipulations, and all measures.

## Study 1

The aim of Study 1 was to explore sources’ emotions while ostracizing as a function of punitive versus defensive motives. After imagining ostracizing for punitive or defensive reasons, participants reported their current levels of anxiety, anger, fear, guilt, happiness, sadness, and pride. As noted above, we predicted that sources motivated by punitive reasons would report higher levels of anger and pride and lower levels of fear, anxiety, sadness, and guilt than sources motivated by defensive reasons.

### Materials and methods

#### Participants

Participants recruited from Amazon’s Mechanical Turk (MTurk) completed the study in exchange for US$1.00. Using the G*Power software [40], we determined that a sample size of 352 was required to detect a small-to-medium effect (*d* = 0.30, the smallest effect of interest) in a two-tailed independent samples *t*-test, with alpha = .05 and power = .80. We recruited 393 participants to account for anticipated data exclusions. Data were excluded per preregistered exclusion criteria: short completion times (*n = 4*), and written responses indicative of a non-serious attempt or failure to follow the instructions (*n* = 17). Thus, analyses were carried out on data from 372 participants (175 females, 197 males, *M*_age_ = 34.92, *SD*_age_ = 10.25). The majority (76.6%) self-reported as White, with other ethnicities selected by fewer than 10% of the sample.

#### Procedure

After providing written informed consent, participants were asked to imagine themselves ostracizing (“giving the silent treatment to”) a friend. They were instructed that this imagined event could be influenced by their own experiences, or it could be entirely made up. In the punitive condition (*n* = 185), participants were asked to imagine that a friend has wronged them in some way and they are now ostracizing their friend to punish them for what they have done. In the defensive condition (*n* = 187), participants were asked to imagine a friend whom they now feel the need to step away or withdraw from and ostracize in order to protect themselves emotionally and/or physically, or to prevent the situation from getting out of hand or escalating. Participants were randomly assigned to conditions and were provided with examples of potential situations that might give rise to their allocated motive.

To enhance engagement with the scenario, participants were asked to type the first name of the person to whom they were imagining ostracizing, which was piped into the remainder of the survey. Next, participants were instructed to write three sentences describing what had led them to ostracize their friend, and three sentences describing how they were excluding and ignoring their friend.

Next, participants reported on emotions experienced while imagining ostracizing. Items assessing happiness, fear, guilt, sadness, and anger were drawn from the Discrete Emotion Scale II (DES-II; [41]). Items assessing pride and anxiety were selected for the purpose of this study. All items (3 items per index) were rated on 9-point scales anchored by *did not feel this at all* and *the most I have ever experienced this*. Items were averaged to create indices for happiness (α = .89), fear (α = .92), guilt (α = .80), sadness (α = .84), anger (α = .86), pride (α = .80), and anxiety (α = .86).

As a check on the manipulation, participants were asked to indicate to what extent they were ignoring the person in the imagined scenario to (a) punish them for something they had done, and (b) to prevent the situation from getting out of hand or escalating. We acknowledge that the manipulation check on defensive motives does not directly capture the self-protection aspect of the defensive motive discussed in previous work [11, 12]. Items were rated on 9-point scales anchored by *this was not at all the reason* and *this was entirely the reason*. Participants provided demographic information before being debriefed.

In both studies we also assessed primary needs (i.e., meaningful existence, belonging, control, and self-esteem) [12]. No significant differences emerged between conditions for any of the primary needs. A summary of the hypotheses, measures, and full results for primary needs is presented in S1 File.

Procedures were carried out in compliance with approval from the University of New South Wales Human Research Ethics Advisory Panel C (#2756).

### Results and discussion

Means, standard deviations, and the results of independent-samples *t*-tests between conditions on motives and experienced emotions are presented in Table 1. Intercorrelations between measures are presented in Table B in S1 File.

**Table 1:** Means, standard deviations, and results of independent samples *t*-tests between punitive and defensive conditions on motives and experienced emotions in Study 1.

	Punitive Condition		Defensive Condition					95% CI^b^	
	*M*	*SD*	*M*	*SD*	*t* ^a^	*p*	*d*	LL	UL
*Motives*									
Punitive	7.97	1.72	4.76	2.92	12.89	< .001	1.34	1.11	1.56
Defensive	3.96	2.83	7.19	2.14	-12.43	< .001	1.29	1.06	1.51
*Emotions*									
Anger	5.23	2.02	4.00	2.19	-5.63	< .001	-0.58	-0.79	-0.38
Anxiety	3.41	2.05	4.15	2.27	3.33	.001	0.34	0.14	0.55
Fear	2.31	1.66	3.02	2.15	3.58	< .001	0.37	0.16	0.57
Guilt	2.76	1.72	3.14	1.87	2.03	.04	0.21	0.01	0.42
Happiness	2.49	1.77	2.55	1.93	0.34	.73	0.03	-0.17	0.24
Pride	3.50	1.84	3.21	2.09	-1.46	.15	-0.15	-0.35	0.06
Sadness	4.30	2.12	4.39	2.18	0.40	.69	0.04	-0.16	0.25

Higher means represent higher levels of a motive/greater intensity of that emotion.

^*a*^*df* = 370. *M* = mean; *SD* = standard deviation. *d* = Cohen’s *d*.

^b^ 95% confidence interval around *d*. LL = lower limit. UL = upper limit.

Participants in the punitive condition reported higher levels of punitive motives and lower levels of defensive motives than participants in the defensive condition. Within conditions, participants in the punitive condition reported higher levels of punitive than defensive motives, *t*(184) = 13.72, *p* < .001, *d* = 1.01, 95%CI [0.83, 1.18]; and participants in the defensive condition reported higher levels of defensive than punitive motives, *t*(186) = -7.69, *p* < .001, *d* = 0.56, 95%CI [0.41, 0.72]. These results demonstrate the paradigm’s efficacy in differentiating motives.

Levels of fear, anxiety, and guilt were lower, and anger higher, in the punitive condition compared to the defensive condition, confirming hypotheses. Sadness and pride did not differ significantly across conditions, against predictions. Happiness also did not differ significantly across conditions, as expected. Broadly, these findings support the notion that source motives govern the emotions experienced by sources while ostracizing: whereas defensive sources are relatively fearful, anxious, and guilty, punitive sources are relatively angry.

Findings for fear, anxiety, and anger align with hypotheses derived from characteristics of the situations thought to give rise to these motives: specifically, that defensive motives stem from situations characterized by threat and low personal control [12, 14], giving rise to fear and anxiety; whereas punitive motives stem from situations characterized by perceived injustices against the self [11, 12] giving rise to anger. However, expected differences in sadness did not emerge, suggesting that helplessness [33] and uncertainty of future negative outcomes [29, 30] characteristic of sadness may not be differentially active in situations that give rise to defensive and punitive ostracism.

Turning to guilt and pride, only the hypothesis regarding lower guilt among punitive relative to defensive sources received support; pride did not differ significantly according to motive. Hypotheses were set in light of the self-conscious nature of these emotions, with the rationale that engaging in punitive ostracism aligns with agentic goals and thus would give rise to lower guilt and higher pride relative to defensive ostracism. However, it may be the case that the moral orientation of these emotions [42, 43] more strongly governed outcomes. It is plausible that ostracizing for defensive reasons violates prescriptive norms associated with social inclusion to a greater degree than ostracizing for punitive reasons, thus giving rise to higher levels of guilt [44]. This is further corroborated by findings that punitive sources are less apologetic about their behavior than sources ostracizing for other motives [2].

Moral aspects of pride, however, are perhaps less influenced by motives in this context. Moral pride is held to be felt upon meeting or exceeding moral standards [42]. Past research has identified that the effort or price associated with enacting positive moral action dictated the intensity of felt moral pride, with more moral pride felt upon actions that required less effort [45]. Moral pride is also responsive to praise and criticism [46], with facilitative and dampening effects, respectively. Extending such findings to sources of ostracism, it may be the case that other contextual features than sources’ motive govern differences in experienced pride–for instance how much effort ostracising entails and/or the praise or criticism received for doing so. This possibility remains to be explored by future research.

In summary, Study 1 offers evidence that source motives govern the emotions experienced by sources while ostracizing: defensive sources are relatively fearful, anxious, and guilty, and punitive sources are relatively angry. Defensive vs. punitive motives do not appear to impact the experience of pride or happiness.

## Study 2

Study 2 aimed to extend Study 1 by investigating the impact of source motive on behavioral intentions, namely intentions to continue ostracizing and to recruit others to join in ostracizing the target. Sources may continue ostracizing to provide opportunity for targets to engage in reparation and may recruit others to provide the source with active support [38]. Some evidence points to motive-driven differences in sources’ future action, particularly in the case of punitive motives. Punitive sources are more likely to continue ostracizing relative to sources ostracizing for other motives (e.g., [38]). Sources motivated by punitive reasons may recruit others to increase the target’s punishment–but also to diffuse the sources’ responsibility for ostracizing the target [14, 25]. Study 2 aimed to provide the first known direct comparison of punitive and defensive sources’ behavioral intentions. In line with previous research [38], we predicted greater intentions to continue ostracizing and to recruit others among participants who imagined ostracizing for punitive reasons relative to defensive reasons.

A second aim of Study 2 was to examine the potential mediating role of emotional experience in giving rise to behavioral intentions. Leveraging theoretical perspectives that emotion drives behavior (e.g., [47, 48]), we investigated the degree to which sources’ emotions mediated the impact of motives on behavioral intentions. In light of high correlations between reported fear and anxiety and between pride and happiness (*r*s = .78) and similar condition-wise results across each pair in Study 1, composite scales of fear/anxiety and happiness/pride were created in Study 2. This approach is recommended to avoid potential multicollinearity in the planned structural equation modelling [49]. We did not set directional hypotheses for indirect effect analyses.

### Materials and methods

#### Participants

We determined that a sample size of 714 was required to detect the smallest effect size that reached statistical significance in Study 1 (i.e., *d* = 0.21) in a two-tailed independent samples *t*-test with alpha level = .05 and power = .80 (G*Power Software; [40]). This “small” effect size was, thus, the minimum detectable with .80 power for the behavioral intention items new to this study. We collected data from 789 participants to allow for potential exclusions. Participants were recruited from MTurk and paid US$1.00. Data were excluded per preregistered exclusion criteria: short completion time (*n* = 14) and noncompliant written responses (*n* = 32). Thus, analyses were carried out on data from 743 participants (365 females, 373 males, 5 identifying as ‘other’; *M*_age_ = 36.64, *SD*_age_ = 11.99). The majority (75.8%) self-reported as White, with other ethnicities selected by fewer than 10% of the sample.

#### Procedure

After providing written informed consent, participants were randomly assigned to imagine ostracizing another person for punitive (*n* = 371) or defensive (*n* = 372) reasons. The procedure of Study 2 replicated Study 1, barring two changes. First, items assessing pride and happiness were combined into an index of positive emotion (α = .91), and items assessing fear and anxiety were combined into an index of fear (α = .93). The remaining emotion measures mirrored Study 1 (α_guilt_ = .79; α_sadness_ = .82; α_anger_ = .87).

Second, participants completed two items assessing behavioral intentions. Participants were asked to imagine that the situation that led them to ostracize was still ongoing, and to indicate the extent to which they would (1) stop versus continue to ostracize their friend (1 = *I would definitely stop giving them the silent treatment*, 9 = *I would definitely continue giving them the silent treatment*), and (2) actively recruit other people to join them in ostracizing their friend (1 = *I definitely would not actively recruit other people to join me*, 9 = *I definitely would actively recruit other people to join me*).

Primary needs were also assessed in Study 2. Condition-wise differences in control needs were nonsignificant as per Study 1. However, in Study 2, participants in the defensive condition reported more fortified belonging, self-esteem, and meaningful existence needs than participants in the punitive condition. A summary of the hypotheses, measures, and full results for primary needs is presented in S1 File.

Procedures were carried out in compliance with approval from the University of New South Wales Human Research Ethics Advisory Panel C (#2756).

### Results and discussion

Means, standard deviations, and the results of independent-samples *t*-tests between conditions on measured constructs are presented in Table 2. Intercorrelations between measures are presented in Table C in S1 File.

**Table 2:** Means, standard deviations, and results of independent samples *t*-tests between punitive and defensive conditions on motives, experienced emotions and behavioral intentions in Study 2.

	Punitive Condition		Defensive Condition					95% CI^b^	
	*M*	*SD*	*M*	*SD*	*t* ^a^	*p*	*d*	LL	UL
*Motives*									
Punitive	7.70	1.89	4.45	2.89	18.12	< .001	1.33	1.17	1.49
Defensive	4.24	2.91	7.17	2.11	-15.72	< .001	1.15	1.00	1.31
*Emotions*									
Anger	5.44	2.14	4.24	2.24	-7.48	< .001	-0.55	-0.69	-0.40
Fear	3.12	1.87	3.82	2.16	4.74	< .001	0.35	0.20	0.49
Guilt	3.02	1.85	3.35	1.94	2.32	.02	0.17	0.03	0.32
Positive Emotion	3.13	1.90	2.96	1.94	-1.19	.23	-0.09	-0.23	0.06
Sadness	4.54	2.16	4.81	2.09	1.73	.08	0.13	-0.02	0.27
*Behavioral Intentions*									
Continue^c^	5.74	2.49	6.76	2.24	5.87	< .001	0.43	0.29	0.58
Recruit^d^	2.61	2.34	2.63	2.23	0.07	.94	0.01	-0.14	0.15

Higher means represent higher levels of a motive/greater intensity of that emotion/higher intention to engage in the behavior.

^a^ *df* = 741. *M* = mean; *SD* = standard deviation. *d* = Cohen’s *d*.

^b^ 95% confidence interval around *d*. LL = lower limit. UL = upper limit.

^c^ Intention to stop vs. continue ostracizing your friend.

^d^ Intention to recruit others to join you in ostracizing your friend.

Participants in the punitive condition reported higher levels of punitive motives and lower levels of defensive motives than participants in the defensive condition. Within condition, participants in the punitive condition reported higher levels of punitive than defensive motives, *t*(370) = 16.87, *p* < .001, *d* = 0.88, 95%CI [0.76, 1.00], and participants in the defensive condition reported higher levels of defensive than punitive motives, *t*(371) = -12.86, *p* < .001, *d* = 0.67, 95%CI [0.55, 0.78]. These results again demonstrate the paradigm’s efficacy in differentiating motives.

Replicating Study 1, levels of fear and guilt were higher, and anger lower, in the defensive condition compared to the punitive condition. Positive emotion and sadness did not differ significantly between conditions, again replicating Study 1.

Contrary to predictions, participants asked to imagine ostracizing for defensive reasons reported higher intentions to continue ostracizing relative to those asked to imagine ostracizing for punitive reasons. Note that detection of this effect was sufficiently powered, given two-tailed tests were specified during sample size determination. Intention to recruit others did not differ significantly as a function of motive condition.

Structural equation modelling carried out in MPlus (Version 7.4; [50]) was deployed to examine the potential mediating role of emotional experience on behavioral intentions. A fully-saturated model was estimated predicting emotions and behavioral intentions from condition (scored as 1 = punitive, 2 = defensive). Emotions were modelled as mediators. Per preregistered model specification, error covariances among the five emotions and, separately, between the two behavioral intentions were estimated. Note that indirect effects do not require the presence of a corresponding total effect [51]. Therefore, despite the nonsignificant effect of condition on intention to recruit others, this outcome was included in the model.

**Fig 1:**
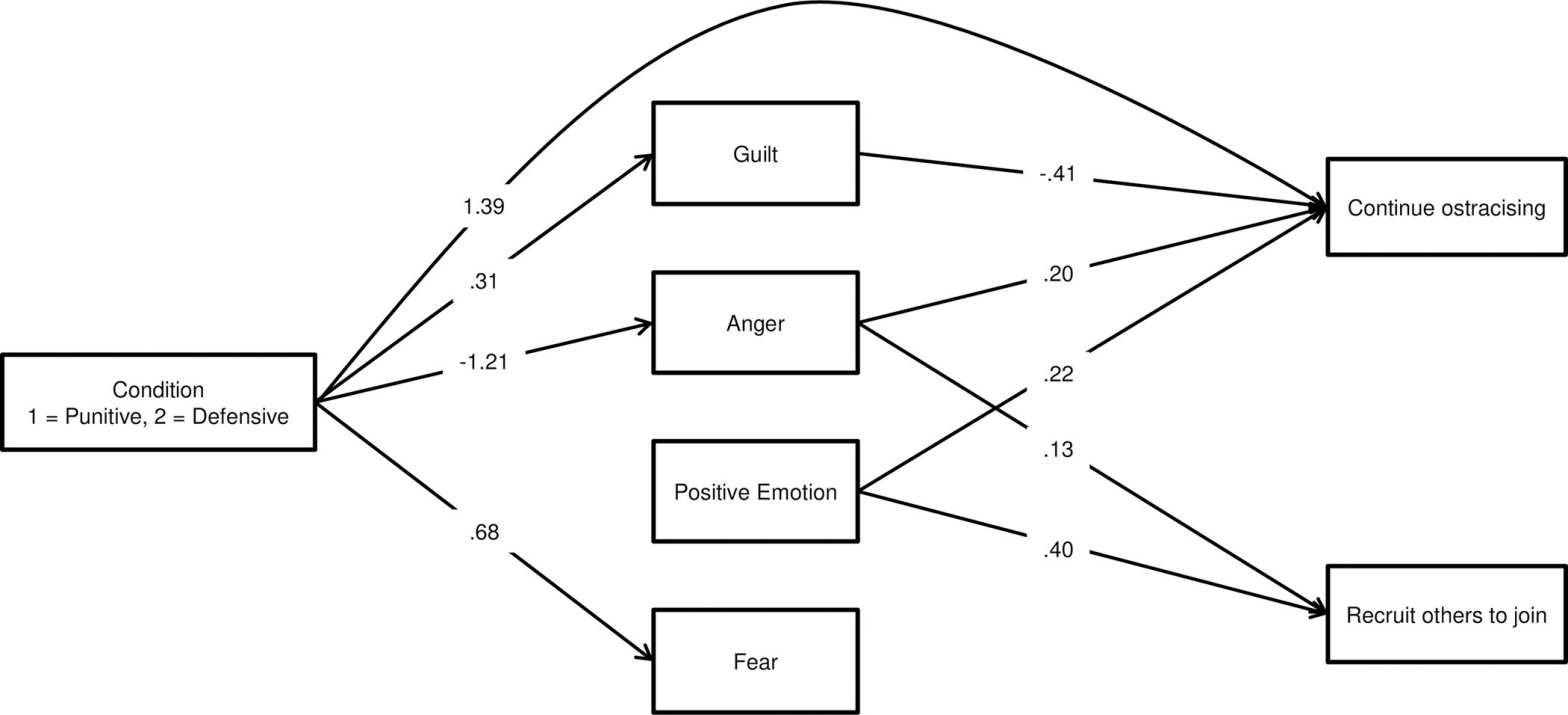
Graphical depiction of the trimmed structural model from Study 2. Unstandardized model estimates are provided. Error variances of endogenous variables and covariances among them are not depicted (error covariance estimates: guilt/anger = .70, guilt/positive emotion = -.23, guilt/fear = 2.53, anger/positive emotion = -.08, anger/fear = 1.51, positive emotion/fear = -.45, continue/recruit = .91); see S1A Fig in S1 File for a version of this figure that includes visual representation of error variances.

Trimming nonsignificant paths (*p* > .05) resulted in sadness being omitted from the model. Fear was retained in the model, but did not significantly predict either behavioral intention. The resulting trimmed model, presented in Fig 1, fit the data well, χ^2^(5) = 5.75, *p* = .33, RMSEA = .01 (90%CI [.00, .05]), CFI > .99, TLI > .99, SRMR = .01 [49].

Significant paths reflected condition comparisons reported above. Further, guilt (-), anger (+), and positive emotion (+) predicted intention to continue ostracizing, and anger (+) and positive emotion (+) predicted intention to recruit others.

Three indirect effects (condition >> guilt >> intention to continue ostracizing, condition >> anger >> intention to continue ostracizing, and condition >> anger >> intention to recruit others) were assessed via bootstrapped confidence intervals. All three confidence intervals excluded zero. Contrasting the observed direct effect, to the extent that ostracizing for defensive versus punitive reasons elicited guilt, intentions to continue ostracizing were lowered (-.13, 95%CI [-.25,-.02]). However, to the extent that ostracizing for punitive versus defensive reasons elicited anger, intentions to continue ostracizing (-.25, 95%CI [-.37, -.15]) as well as to recruit others (-.15, 95%CI [-.26, -.07]) were enhanced. These effects emerged while controlling for the positive impact of experienced positive emotion on behavioral intentions; regardless of motive, higher levels of experienced positive emotion in the ostracism episode led participants to desire continuing to ostracize and to recruit others.

This nuanced picture of the impact of source motive on behavioral intentions in light of emotional experience highlights the importance of examining emotions in this context. Generally, these findings support predictions regarding the mediating role of emotional experience in guiding behavior (e.g., [47, 48]). Further, the effects of guilt and anger align with past work regarding these emotions (e.g., guilt: [52] anger: [53, 54]).

## Internal meta-analyses

To establish a more robust estimate of observed effects, internal meta-analyses were conducted on emotion measures across Studies 1 and 2 using the processes detailed by Goh, Hall, and Rosenthal [55]. Fixed effects analyses with inverse variance weighting were employed. To enable comparability, indices of positive emotion (α = .90) and fear (α = .92) were computed for Study 1. Results appear in Table 3. Significant, non-zero effects emerged for fear, anger, and guilt: defensive motives resulted in higher fear and guilt and lower anger relative to punitive motives. Effect sizes ranged from small (guilt) to medium (fear and anger). Differences in positive emotion and sadness were smaller and nonsignificant.

**Table 3:** Results of internal meta-analyses of experienced emotions across Studies 1 and 2.

		95% CI^a^			
	*d*	LL	UL	*Z*	*p*
Anger	-0.56	-0.68	-0.44	-9.17	< .001
Fear	0.44	0.33	0.56	7.31	< .001
Guilt	0.19	0.07	0.30	3.104	.002
Positive Emotion	-0.08	-0.20	0.04	-1.36	.175
Sadness	0.10	-0.02	0.22	1.65	.100

^*a*^*d* = Cohen’s *d*. LL = lower limit of 95% confidence interval. UL = upper limit of 95% confidence interval.

## General discussion

The current studies are the first to compare the psychological experience of ostracizing for two common motives: punitive and defensive [2, 16, 17]. We deployed a novel paradigm designed to induce the experience of ostracizing for these motives to assess emotional experiences (Studies 1 & 2) and behavioral intentions (Study 2). These studies reveal new insights into the nature of ostracism sources’ experience, highlighting the crucial role of the specific motives in guiding emotions and behavior.

Results highlight the importance of motive when considering sources’ emotional experience while ostracizing. Across both studies and supported by an internal meta-analysis, ostracizing for defensive reasons was associated with higher levels of guilt and fear and lower levels of anger compared to ostracizing for punitive reasons. Neither sadness nor positive emotion (pride or happiness) differed significantly according to motive in either study.

Moreover, two emotions were linked to distinct behavioral intention profiles in Study 2: guilt and anger. To the extent that punitive sources experienced anger relative to defensive sources, they expressed greater intentions to continue ostracizing the target and to recruit others to join in ostracizing the target. To the extent that defensive sources experienced guilt relative to punitive sources, they reported reduced intentions to continue ostracizing the target. Interestingly, in the absence of guilt, defensive sources carried higher intentions to continue ostracizing relative to punitive sources. This pattern of inconsistent mediation (i.e., effects of conflicting direction in indirect effects model; MacKinnon, Fairchild, & Fritz, 2007) is worthy of replication and further exploration. One explanation for why defensive motives lead to desire for continuation of ostracism more so than punitive motives is that unresolved punitive motives may result in sources considering other actions rather than continuing to ostracise. Wirth and Wesselmann [6] describe ostracism driven by punitive motives as a ‘social influence tactic’–in this logic, a source motivated by punitive reasons might tactically decide to pursue other actions that would correct the behavior that instigated the ostracism episode. Sources motivated by defensive reasons, on the other hand, may not feel as empowered to seek other routes of action, or perhaps other actions may be perceived as even less likely to satisfy the situation than continued ostracism. This possibility is worthy of attention in future research.

Broadly, findings involving guilt and anger align with current understanding regarding how these emotions shape antisocial behavior. Specifically, guilt motivates self-regulation aimed at mitigating actions that gave rise to its experience, including regulating aggression [44, 56]. Anger, in contrast, motivates action with the aim of gaining control or retribution [57], such as aggression [53, 58]. Interestingly, the link between source anger and continued antisocial behavior toward the target mirrors the link between target anger and antisocial behavior, particularly toward sources (see [59–61]). Moreover, the patterns for guilt that emerged here are consistent with findings of sources’ increased willingness to engage prosocially to make amends for having ostracized to the extent that ostracism was perceived as immoral (and thus inhumane; [62]). Generally, it appears that anger felt by either party in an ostracism episode prompts continuation of the behavior, whereas guilt felt by a source serves as a mechanism via which the episode might end. This conclusion aligns well with perspectives of guilt and anger as moral emotions that operate in contexts where moral social tenets have been violated (e.g., [63]).

Whereas pride and happiness were not differentially elicited by punitive versus defensive motives, participants reporting higher levels of these emotions were indeed more inclined to continue ostracizing and to recruit others to join in ostracizing the target. Considering sources’ overarching goal to ostracize, these findings are consistent with those linking pride [39] and positive affect [64] with goal pursuit. Perhaps efficacious ostracism is a source of positive emotion in an otherwise negative context.

Our focus on sources’ emotions while engaging in ostracism, and the subsequent effect of those emotions on behavior, paves the way for development of interventions that might shorten or end ostracism episodes. Namely, interventions that target elevating defensive sources’ guilt and attenuating punitive sources’ anger may curb continued ostracism. Techniques drawn from the emotion regulation literature (e.g., reappraisal to reduce anger; [65]) may be particularly potent.

The current research focused on sources’ emotions while ostracizing, including those that may have carried over from the event that prompted the ostracism (e.g., anger among punitive sources and fear/anxiety among defensive sources) as well as those that might arise while ostracizing (e.g., guilt felt because of having ostracized). Future research is needed to investigate emotions on other timescales–for instance those in anticipation of future ostracism and upon recall of past ostracism–and how they might impact behavior. Pinning down the temporal sequelae at hand within an ostracism episode is also of high interest. It is likely that unfolding information about the efficacy of ostracism and/or the target’s response would impact emotions and downstream behavioral intentions. An ideal, though highly resource intensive, design would measure motives, emotions, and behavioral intentions live as an ostracism episode unfolds to disentangle the causal sequences at play. Data collected from both sources and targets would be of high value.

The current research has implications for theoretical models of ostracism. Williams’ [13] temporal need-threat model posits that affect is a reflexive and immediate response to ostracism, whereas appraising the motives or reasons for the ostracism comes online after reflecting on the act. This model would predict that experienced emotion should not be impacted by the motive for ostracism. However, our finding that sources’ emotional experiences differ as a function of the motives for ostracizing suggests that the need-threat model may need modification. Whereas targets might experience ostracism immediately without considering underpinning reasons, inherent in sources’ experience are their motives.

This research also points to the importance of sources’ sense of justification for engaging in ostracism. Punitive sources’ heightened anger and decreased guilt is compatible with a stronger sense of justification relative defensive sources. One basis of justification may come from attributions of responsibility [66]. Indeed, punitive motives are associated with attributing responsibility to the target [2], with punitive sources expecting targets to apologize for their past behavior [38]. Post-hoc victim blaming, which serves to rationalize ostracism and enable sources to maintain a positive self-concept [6], may be invoked more among punitive sources. While defensive sources may attribute responsibility to the target (e.g., the target is being threatening), they may also attribute responsibility to themselves (e.g., they cannot defend themselves), to the situation (e.g., it is getting out of hand), or a combination of these factors. This ambiguity may lead to lower justification levels among defensive sources. Direct assessment of sources’ sense of justification as a proximate mechanism of the observed effects will be an important avenue for future research.

The paradigm we developed expands the methodological toolkit for studying source experiences. It is the first to successfully differentiate between specific source motives, building on a growing list of paradigms that broadly elicit motivated ostracism (see [4] for a review). Further, this paradigm offers enhanced ecological validity, enabling participants to draw from their own real-world ostracism experiences to imagine ostracizing for defensive or punitive reasons, thus moving away from the contrived settings offered by currently available laboratory paradigms (for further discussion see [14]). We are excited that this paradigm could be adapted to investigate the effects of ostracizing for other motives (e.g., role-prescribed; [11, 12]). This paradigm also paves the way for investigating source motives in workplace contexts–an area of recent and increasing research focus (e.g., [15, 67–70]). Focusing on motives may shed light on contextual variables that drive workplace ostracism, and the corresponding emotional and behavioral consequences of those motives. Moreover, in light of documented workplace “incivility spirals,” whereby acts of incivility can escalate into more intense forms of antisocial and/or aggressive behaviors [71–73], this paradigm can be used to identify to whom (based on motive) and when de-escalation approaches might be best deployed.

Several limitations should be acknowledged. First, we only considered a small range of potential behaviors in which sources might engage and only measured the selected behaviors via single items. It may be the case that guilt also prompts prosocial behavior (e.g., [52, 74]) and self-punishment [75], especially among defensive sources. Further, it will be important for future research to examine behaviors associated with fear and anxiety in this context, given that they were not predictive of either behavior examined in Study 2. At first glance, this null link is perplexing from the perspective that ostracism is a form of avoidance, and that fear and anxiety both prompt avoidance. Sources’ fear and anxiety may prompt other forms of avoidance, such as risk aversion [76], especially if ostracism is not effective in reducing fear and anxiety that prompted ostracism in the first place. This possibility remains to be tested in future research. Moreover, mechanisms underlying defensive sources’ desire to continue ostracizing more so than punitive sources (i.e., the remaining direct effect in the model) need to be established.

Second, while behavioral intentions are held to be immediate antecedents to actual behavior [77, 78], future research should assess sources’ actual behavior. Several paradigms that assess targets’ antisocial and prosocial behaviors (e.g., [79]) could adapted to study source behavior in the laboratory (see also [80]). Experience sampling may also be useful in building a comprehensive understanding of how source motives both give rise to and are impacted by emotional experience, and how these relate to source behavior in everyday life (see [2]).

Third, these studies were limited in their consideration of punitive and defensive motives. While these were selected based on their relative prevalence [2, 16, 17] and their relatively autonomous nature, other motives may guide distinct source experience. For example, ostracizing for oblivious reasons (i.e., simply not noticing the target) may be less emotionally-provoking for sources given its inadvertent nature [2, 11]. Future research may also differentiate between defensive ostracism as a form of self-protection and defensive ostracism to de-escalate a situation via manipulation and/or measurement. Further, instances of ostracism may be motivated by several reasons at once, such as ostracizing to punish the target *and* to de-escalate the situation [17, 38]. Future research should expand the scope of motives considered and examine the potential for sources’ mixed motives.

Finally, the current research instructed participants to imagine a situation in which they ostracized for different motives. This hypothetical vignette approach was utilized so that participants who could not recall an ostracism episode that suited their allocated motive could instead engage their imagination to fulfil the task (see [81, 82], for similar methodologies). However, the realism of the imagined scenarios is unclear. Assuaging concerns on this point, as reported in S1 File, participants in the pilot study reported drawing from personal experience to complete the paradigm, though the degree to which they did so varied according to motive (with defensive more than punitive). Future research may examine the rates at which participants are able to recall past episodes of punitive or defensive ostracism, and whether emotional experiences and/or behavioral intentions vary as a function of imagined or recalled ostracism.

## Conclusions

The current research examined sources’ emotional experience and behavioral intentions as a function of punitive versus defensive motives, achieved via a novel induction paradigm. Results across two studies robustly support the conclusion that when examining ostracism source experiences, motives do indeed matter. Punitive sources report greater anger and less guilt and fear than defensive sources. Moreover, sources’ guilt and anger are linked to behavioral inclinations in the ostracism context. Specifically, to the extent that punitive sources experience anger, they are more inclined to continue ostracizing the target and to recruit others to join in ostracizing the target. In contrast, to the extent that defensive sources experience guilt, they are less inclined to continue ostracizing the target. It is our hope that this research will inspire further work into the complex psychological and behavioral consequences of performing this social act.

## Supporting information

S1 File Results from pilot study, primary needs, and intercorrelation analyses. (DOCX)
