# Supplementary material for: Motives matter: The psychological experience of ostracizing among sources
Source: PLoS One. 2024 May 31;19(5):e0303510. doi: 10.1371/journal.pone.0303510 (PMC11142671; doi:10.1371/journal.pone.0303510)
Supplement: S1 File — (DOCX) [file pone.0303510.s001.docx]

**S1 Supporting Information. Results from pilot study, primary needs, and intercorrelation analyses (Iannuzzelli, Gonsalkorale, & Williams)**

This Supporting Information includes:

- Report of a Pilot Study testing a paradigm that induces the experience of ostracising for different motives
- Details on primary needs (Studies 1 and 2)
- Intercorrelations between measures (Studies 1 and 2)
- Structural modelling results figure, including visual depiction of error covariances (Study 2)

**Pilot Study**

As no prior study has directly compared the consequences of ostracizing for different motives, the aim of the Pilot Study was to evaluate the effectiveness of an experimental paradigm that induces the experience of ostracizing for different motives. We adapted an autobiographical recall paradigm used in prior ostracism research (e.g., Sommer, Williams, Ciarocco, & Baumeister, 2001; Zhong & Leonardelli, 2008) to hypothetical vignettes capturing specific motives. The move from autobiographical recall to hypothetical vignette was intended to ensure participants could imagine an ostracism experience in which they ostracized for the specific motive in the case that they could not draw on a specific prior memory. Previous research has utilized hypothetical vignette paradigms to investigate ostracism, finding that imagining an ostracism episode inspires comparable responses to experiencing ostracism (Chen & Williams, 2012; Giesen & Echterhoff, 2018; Williams, Shore, & Grahe, 1998).

In the Pilot Study, participants were instructed to imagine a scenario in which they ostracized another individual for either punitive or defensive reasons. After writing about the scenario, participants completed measures assessing punitive and defensive motives. In accordance with the intended manipulation of motives, we expected differences between conditions such that participants in the defensive condition would report greater defensive and less punitive motives than participants in the punitive condition. We also expected within-condition differences such that defensive motives would be higher than punitive motives within the defensive condition, and the reverse in the punitive condition. Given the novelty of the paradigm, we also explored characteristics of participants’ experience of it – including the degree to which they drew from personal experience, the vividness of the imagined scenario, and the ease with which they imagined the scenario. Hypotheses regarding these latter variables were not set.

**Method**

**Participants.** Participants recruited from Amazon’s Mechanical Turk (MTurk) completed the study in exchange for US$0.75. An a priori power analysis conducted in G*Power (Faul, Erdfelder, Lang, & Buchner, 2007) revealed that a sample size of *N* = 128 was required to detect a medium-sized effect (*d* = 0.50) via an independent-samples *t*-test, with an alpha level of .05 and power of .80. We chose a conservative medium-sized effect due to the novel nature of the study. We collected data from 141 participants in anticipation of a 10% exclusion rate. Data from five participants were excluded based on screening participants’ written responses (i.e., participants did not write anything, written content was considered a non-serious attempt, or participants did not follow the instructions). Thus, analyses were carried out on data from 136 participants (59 females, 77 males, *M*_age_ = 34.26, *SD*_age_ = 10.95), who self-reported as White (71.3%), African-American (9.6%), Hispanic/South American/Central American (2.2%), North East Asian (6.6%), South East Asian (3.7%), Native American (0.7%), African (0.7%), with 5.1% indicating ‘Other’ or selecting multiple ethnicities.

**Procedure.** After providing consent, participants were asked to imagine themselves giving the silent treatment to a friend. They were instructed that this imagined event could be influenced by their own experiences, or it could be entirely made up. In the punitive condition (*n* = 63), participants were asked to imagine that a friend has wronged them in some way and they are now ostracizing their friend to punish them for what they have done. In the defensive condition (*n* = 73), participants were asked to imagine a friend who they now feel the need to step away or withdraw from and ostracize in order to protect themselves emotionally and/or physically, or to prevent the situation from getting out of hand or escalating. Participants were randomly assigned to conditions and were provided with examples of potential situations that might give rise to their allocated motive.

To enhance engagement with the scenario, participants were asked to type the first name of the person to whom they were imagining giving the silent treatment. Next, participants were instructed to write three sentences describing what had led them to give their friend the silent treatment, and three sentences describing how they were excluding and ignoring their friend.

As a check on the manipulation, participants were asked to indicate to what extent they were ignoring the person in the imagined task to (a) punish them for something they had done, and (b) to prevent the situation from getting out of hand or escalating. Items were rated on a 9-point scale anchored by *this was not at all the reason* and *this was entirely the reason*.

As a further check on how participants engaged with the manipulation, participants were asked to indicate to what extent the situation they imagined corresponded to an experience they have had in real-life (1 = *I did not draw from personal experience at all*, 9 = *I drew directly from personal experience to a great degree*), how vividly they had imagined the situation during the task (1 = *not at all vividly*, 9 = *extremely vividly*), and how easy it was for them to imagine the situation (1 = *not at all easy*, 9 = *extremely easy*). Before being debriefed, participants were asked to provide demographic information.

**Results and Discussion**

Self-reported punitive motives and defensive motives were negatively correlated, *r*(134) = -.54, *p* < .001. As expected, and supporting the efficacy of the manipulation, participants in the punitive condition (*M* = 7.95, *SD* = 1.68) reported higher levels of punitive motives than participants in the defensive condition (*M* = 5.49, *SD* = 2.86), *t*(134) = 5.99, *p* < .001, *d* = 1.03, 95%CI [0.67, 1.39]. Further, participants in the defensive condition (*M* = 7.15, *SD* = 2.02) reported higher levels of defensive motives than participants in the punitive condition (*M* = 3.68, *SD* = 2.68), *t*(134) = 8.60, *p* < .001, *d* = 1.48, 95%CI [0.10, 1.86].

Results of the comparisons of motive levels within conditions were also as expected: participants in the punitive condition reported more punitive than defensive motives, *t*(62) = 9.27, *p* < .001, *d* = 1.17, 95%CI [0.84, 1.49]; and participants in the defensive condition reported more defensive than punitive motives, *t*(72) = 3.42, *p* = .001, *d* = 0.40, 95%CI [0.16, 0.64]. Both sets of results support the efficacy of our adapted paradigm inducing the experience of ostracizing for defensive versus punitive motives, with motives varying both within and across conditions.^[[1]](#footnote-1)^

Analysis of the remaining three items revealed that participants in the defensive condition (*M* = 7.16, *SD* = 2.30) reported that they drew from personal experiences significantly more than participants in the punitive condition (*M* = 6.13, *SD* = 2.93), *t*(134) = 2.31, *p* = .02, *d* = 0.40, 95% CI [0.06, 0.74]. This appears to contradict previous studies documenting that punitive reasons are the most common motive raised in recall paradigms that do not manipulate motive (e.g., Poulsen & Carmon, 2015; Sommer et al., 2001). However, one interpretation of this result is that participants may be more comfortable recalling episodes of defensive than punitive ostracism. Nonetheless, it is worth noting that the mean levels on this item were above the scale midpoint in both conditions, suggesting that participants were more likely than not to draw from personal experience when imagining a hypothetical ostracism episode for either motive. No significant differences emerged between conditions for vividness or ease of imagination, *p*s > .47, *d*s < 0.12.

**Primary Needs**

In Studies 1 and 2, after engaging in the source motives paradigm participants were instructed to continue imagining giving their friend the silent treatment while responding to 12 items that assessed primary needs (Williams, 2001), detailed below. These items were completed prior to the emotion items and, in Study 2, the behavioral intention items. Results of analyses of the emotion measures and behavioral intention items are reported in the main text.

We pre-registered a priori predictions regarding the impact of source motive on primary needs (available on the OSF site: https://osf.io/632ye/?view_only=82f7871fce02441d98cbbe93b649a860). In Study 1, we predicted that participants in the punitive condition would report more fortified belonging and control than participants in the defensive condition. This prediction was founded in the theoretical premise that ostracizing to punish the target, who is believed to deserve to be punished for something they have done, may lead to a greater sense of power and control than ostracizing to protect oneself (Zadro et al., 2017). Additionally, the act of withdrawing associated with defensive ostracism (Williams, 2001) suggests social disconnection that may undermine belonging, a theme not present in the more retributive use of punitive ostracism. Theoretical differences between punitive and defensive motives do not give rise to differential predictions for self-esteem or meaningful existence needs; as such, these were not expected to differ significantly between conditions.

Examination of the literature yielded one study that investigated the impact of specific ostracism motives on sources’ primary needs. Nezlek et al. (2015) found that relative to other motives, ostracizing for punitive reasons fortified sources’ sense of control, self-esteem, and meaningful existence and depleted their sense of belonging. In contrast, ostracizing for defensive reasons only fortified sources’ sense of control and depleted belonging. However, this study did not directly compare reported primary needs between punitive and defensive sources.

In both Studies 1 and 2, the four primary needs were assessed using a questionnaire adapted from Williams (2001), consisting of 12 items (3 items per ‘need’) assessing belonging (e.g., “I felt rejected”), self-esteem (e.g., “I felt liked”), meaningful existence (e.g., “I felt invisible”), and control (e.g., “I felt powerful”). All items were rated on 9-point scales anchored by *did not feel this at all* and *the most I have ever experienced this*. Items were reverse-scored as necessary and averaged to form subscales of belonging (Study 1: α = .74; Study 2: α = .74), self-esteem (Study 1: α = .80; Study 2: α = .77), meaningful existence (Study 1: α = .82; Study 2: α = .80), and control (Study 1: α = .71; Study 2: α = .72), where higher scores indicated more fortified needs.

Means, standard deviations, and the results of independent-samples *t*-tests between conditions on primary needs in Studies 1 and 2 are presented in Table A. In Study 1, contrary to predictions, levels of belonging and control did not significantly differ between conditions. In line with our predictions, levels of self-esteem and meaningful existence did also not significantly differ across conditions. Thus, the results of Study 1 only partially supported our predictions.

Based on our findings in Study 1, our pre-registered predictions for Study 2 were that there would be no significant differences in primary needs across conditions. As can be seen in Table A, in line with Study 1, levels of control did not significantly differ between conditions. However, contrary to expectations, participants in the defensive condition reported more fortified belonging, self-esteem, and meaningful existence than participants in the punitive condition, though effect sizes were modest.

As the first study to experimentally compare the consequences of ostracizing for punitive versus defensive motives, predictions were based on theoretical differences between these motives. However, findings do not support these predictions. Results suggest that further research is needed to clarify the nature of the impact of source motives on primary needs.

**Intercorrelations Between Measures**

In Tables B and C, we report intercorrelations between measures (manipulation checks, emotions, primary needs, and, in Study 2, a dummy coded condition variable and behavioural intentions) in Studies 1 and 2, respectively. We did not preregister these analyses; they were performed upon reviewer request.

Table A

*Means, Standard Deviations, and Results of Independent Samples t-tests between Punitive and Defensive Conditions on Primary Needs in Studies 1 and 2*

|  | Punitive Condition | |  | Defensive Condition | |  |  |  | 95% CI^2^ | |
| --- | --- | --- | --- | --- | --- | --- | --- | --- | --- | --- |
|  | *M* | *SD* |  | *M* | *SD* | *t^1^* | *p* | *d* | LL | UL |
| **Study 1** |  |  |  |  |  |  |  |  |  |  |
| Belonging | 5.59 | 2.12 |  | 5.65 | 2.06 | 0.24 | .81 | 0.03 | -0.18 | 0.23 |
| Self-Esteem | 3.94 | 1.90 |  | 4.19 | 1.86 | 1.27 | .20 | 0.13 | -0.07 | 0.34 |
| Meaningful Existence | 6.44 | 2.05 |  | 6.59 | 2.03 | 0.72 | .47 | 0.07 | -0.13 | 0.28 |
| Control | 5.39 | 1.82 |  | 5.51 | 1.84 | 0.61 | .54 | 0.07 | -0.14 | 0.27 |
| **Study 2** |  |  |  |  |  |  |  |  |  |  |
| Belonging | 5.33 | 2.15 |  | 5.66 | 2.07 | 2.13 | .03 | 0.16 | 0.01 | 0.30 |
| Self-Esteem | 3.92 | 1.92 |  | 4.24 | 1.98 | 2.22 | .03 | 0.16 | 0.02 | 0.31 |
| Meaningful Existence | 6.19 | 2.09 |  | 6.65 | 1.94 | 3.14 | .002 | 0.23 | 0.08 | 0.37 |
| Control | 5.37 | 1.97 |  | 5.45 | 1.82 | 0.60 | .55 | 0.04 | -0.10 | 0.19 |

*^1^*Study 2 *df* = 370; Study 3 *df* = 741. *M* = mean; *SD* = standard deviation. *d* = Cohen’s *d*. ^2^ 95% confidence interval around *d*. LL = lower limit. UL = upper limit. Higher means represent more fortified needs.

Table B

*Correlations between measures in Study 1*

|  | 1. | 2. | 3. | 4. | 5. | 6. | 7. | 8. | 9. | 10. | 11. | | 12. |
| --- | --- | --- | --- | --- | --- | --- | --- | --- | --- | --- | --- | --- | --- |
| 1. Punitive Motives |  |  |  |  |  |  |  |  |  |  |  | |  |
| 2. Defensive Motives | -.602^**^ |  |  |  |  |  |  |  |  |  |  | |  |
| 3. Happiness | .042 | -.008 |  |  |  |  |  |  |  |  |  | |  |
| 4. Anxiety | -.211^**^ | .148^**^ | .009 |  |  |  |  |  |  |  |  | |  |
| 5. Fear | -.227^**^ | .171^**^ | .137^**^ | .782^**^ |  |  |  |  |  |  |  | |  |
| 6. Pride | .130^*^ | -.077 | .777^**^ | -.075 | .004 |  |  |  |  |  |  | |  |
| 7. Guilt | -.200^**^ | .088 | .190^**^ | .647^**^ | .639^**^ | .057 |  |  |  |  |  | |  |
| 8. Sadness | -.084 | .034 | -.188^**^ | .575^**^ | .454^**^ | -.282^**^ | .526^**^ |  |  |  |  | |  |
| 9. Anger | .291^**^ | -.211^**^ | .003 | .221^**^ | .214^**^ | .103^*^ | .150^**^ | .289^**^ |  |  |  | |  |
| 10. Belonging | -.017 | .015 | .023 | -.342^**^ | -.274^**^ | .108^*^ | -.397^**^ | -.567^**^ | -.202^**^ |  |  | |  |
| 11. Self Esteem | -.012 | .015 | .483^**^ | -.175^**^ | -.082 | .581^**^ | -.074 | -.259^**^ | -.050 | .248^**^ |  | |  |
| 12. Meaningful Existence | -.038 | .002 | -.113^*^ | -.288^**^ | -.291^**^ | -.013 | -.409^**^ | -.452^**^ | -.171^**^ | .764^**^ | .153^**^ | |  |
| 13. Control | .083 | -.049 | .299^**^ | -.079 | -.084 | .456^**^ | -.099 | -.216^**^ | .048 | .280^**^ | .557^**^ | | .224^**^ |
| **. Correlation is significant at the 0.01 level (2-tailed). | | | | | | | | | | | |  |  |
| *. Correlation is significant at the 0.05 level (2-tailed). | | | | | | | | | | | |  |  |

Table C

*Correlations between measures in Study 2*

|  | | 1. | 2. | 3. | 4. | 5. | 6. | 7. | 8. | 9. | 10. | 11. | 12. | 13. |  |
| --- | --- | --- | --- | --- | --- | --- | --- | --- | --- | --- | --- | --- | --- | --- | --- |
| 1. Condition | |  |  |  |  |  |  |  |  |  |  |  |  |  |  |
| 2. Punitive Motives | | -.554^**^ |  |  |  |  |  |  |  |  |  |  |  |  |  |
| 3. Defensive Motives | | .500^**^ | -.491^**^ |  |  |  |  |  |  |  |  |  |  |  |  |
| 4. Positive Emotion | | -0.043 | .158^**^ | .024 |  |  |  |  |  |  |  |  |  |  |  |
| 5. Fear | | .164^**^ | -.192^**^ | .168^**^ | -.103^**^ |  |  |  |  |  |  |  |  |  |  |
| 6. Guilt | | .075^*^ | -.109^**^ | .070 | -.043 | .658^**^ |  |  |  |  |  |  |  |  |  |
| 7. Sadness | | .077^*^ | -.087^*^ | .046 | -.340^**^ | .577^**^ | .503^**^ |  |  |  |  |  |  |  |  |
| 8. Anger | | -.258^**^ | .289^**^ | -.159^**^ | .014 | .261^**^ | .140^**^ | .346^**^ |  |  |  |  |  |  |  |
| 9. Belonging | | .072^*^ | -.008 | .015 | .198^**^ | -.387^**^ | -.375^**^ | -.503^**^ | -.255^**^ |  |  |  |  |  |  |
| 10. Self Esteem | | .093^*^ | .012 | .107^**^ | .590^**^ | -.191^**^ | -.178^**^ | -.230^**^ | -.027 | .336^**^ |  |  |  |  |  |
| 11. Meaningful Existence | | .117^**^ | -.054 | .060 | .022 | -.311^**^ | -.351^**^ | -.353^**^ | -.169^**^ | .721^**^ | .221^**^ |  |  |  |  |
| 12. Control | | .031 | .080^*^ | .105^**^ | .431^**^ | -.116^**^ | -.127^**^ | -.202^**^ | .095^**^ | .290^**^ | .596^**^ | .303^**^ |  |  |  |
| 13. Continue Intentions | | .211^**^ | -.048 | .206^**^ | .171^**^ | -.135^**^ | -.275^**^ | -.137^**^ | .057 | .207^**^ | .232^**^ | .231^**^ | .236^**^ |  |  |
| 14. Recruit Intentions | | .003 | .134^**^ | .022 | .328^**^ | .056 | .038 | -.051 | .146^**^ | .056 | .218^**^ | .011 | .181^**^ | .239^**^ |  |
|  | *Note*. Condition reflects a dummy coded variable with 1 = Punitive and 2 = Defensive.  **. Correlation is significant at the 0.01 level (2-tailed). | | | | | | | | | | | | | | |
|  | *. Correlation is significant at the 0.05 level (2-tailed). | | | | | | | | | | | | | | |


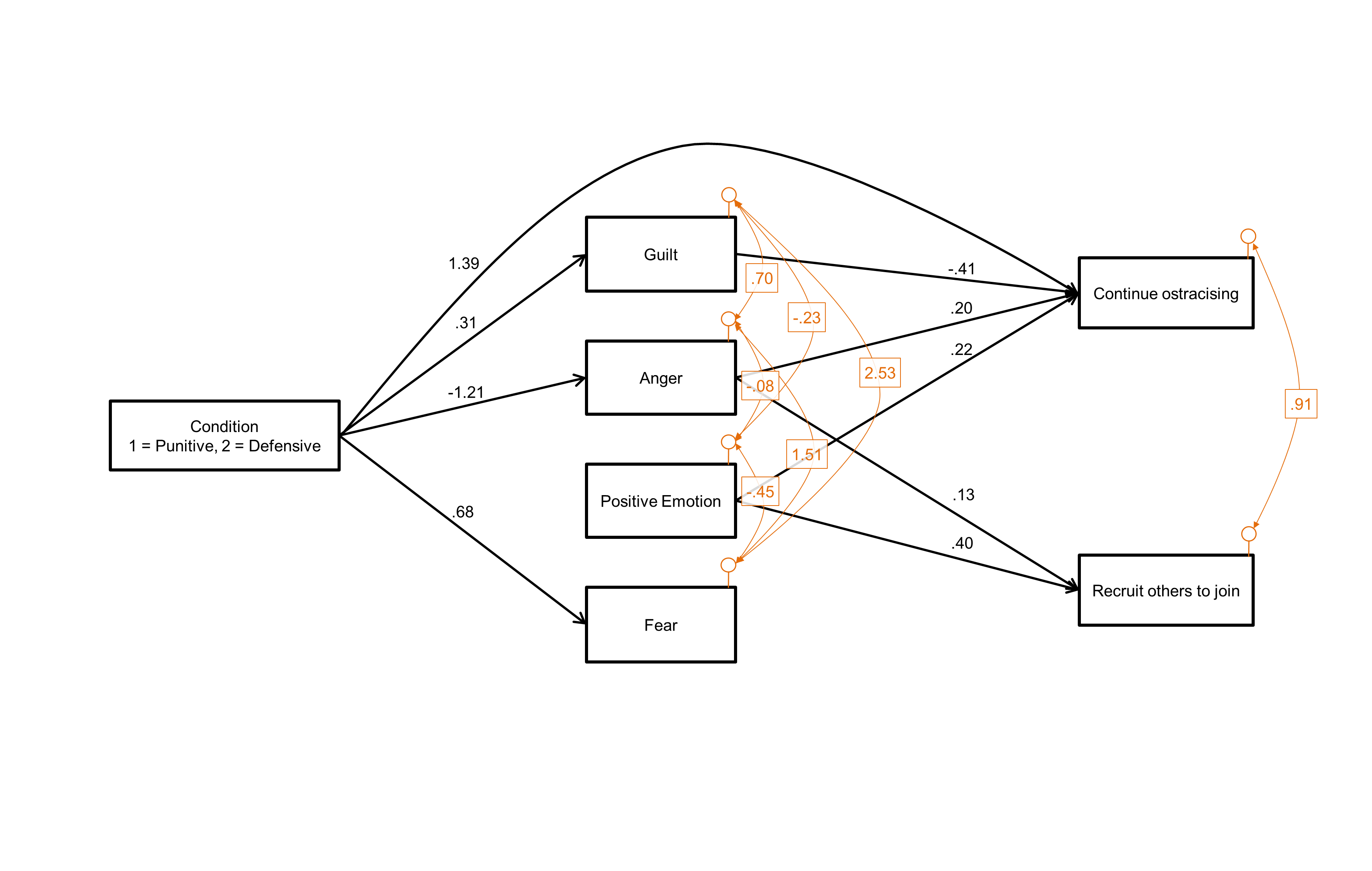
 **Fig A. Graphical depiction of the trimmed structural model from Study 2.** Unstandardized model estimates are provided. Error variances of endogenous variables and covariances among them are depicted in orange.

**References**

Chen, Z., & Williams, K. D. (2012). Imagined future social pain hurts more now than imagined future physical pain. *European Journal of Social Psychology, 42*(3), 314-317.

Faul, F., Erdfelder, E., Lang, A. G., & Buchner, A. (2007). G*power 3: A flexible statistical power analysis program for the social, behavioural, and biomedical sciences. *Behaviour Research Methods, 39*, 175–191.

Giesen, A., & Echterhoff, G. (2018). Do I really feel your pain? Comparing the effects of observed and personal ostracism. *Personality and Social Psychology Bulletin, 44*(4), 550-561.

Nezlek, J. B., Wesselmann, E. D., Wheeler, L., & Williams, K. D. (2015). Ostracism in everyday life: The effects of ostracism on those who ostracize. *Journal of Social Psychology, 155*(5), 432-451. doi:10.1080/00224545.2015.1062351

Poulsen, J. R., & Carmon, A. F. (2015). Who would do that? A theory-based analysis of narratives of sources of family ostracism. *The Journal of Social Psychology*, *155*(5), 452-470.

Sommer, K. L., Williams, K. D., Ciarocco, N. J., & Baumeister, R. F. (2001). When silence speaks louder than words: Explorations into the intrapsychic and interpersonal consequences of social ostracism. *Basic and Applied Social Psychology*, *23*(4), 225-243.

Williams, K. D. (2001). Ostracism: A review of the literature. In K. D. Williams (Ed.), *Ostracism: The Power of Silence* (pp. 7-15). New York: Guildford Press.

Williams, K. D., Shore, W. J., & Grahe, J. E. (1998). The silent treatment: Perceptions of its behaviors and associated feelings. *Group Processes and Intergroup Relations, 1*, 117- 141.

Zadro L., Godwin A., Svetieva E., Sethi N., Iannuzzelli R., Gonsalkorale K. (2017). Creating the silence: Ostracism from the perspective of the source. In Williams K. D., Nida S. (Eds.), Ostracism, Exclusion, and Rejection (pp. 131–145). New York, NY: Psychology Press.

Zhong, C. B., & Leonardelli, G. J. (2008). Cold and lonely: Does social exclusion literally feel cold?. *Psychological Science*, *19*(9), 838-842.

1. Post-hoc exploratory analyses found that participants’ ratings of whether they had imagined ostracizing for punitive or defensive motives were significantly negatively correlated overall (*r* = -.54, *p* < .001), and separately within conditions (punitive: *r* = -.38, *p* = .002; defensive: *r* = -.43, *p* < .001) suggesting that participants were unlikely to report mixed punitive and defensive motives. [↑](#footnote-ref-1)
